# Congenital intrathoracic accessory spleen is a very rare trick of nature: a case report

**DOI:** 10.1186/s13019-020-01270-4

**Published:** 2020-08-31

**Authors:** Mohammed O. Suraju, Nicole Peyton, Brian Mooers, Chris Jensen, Joel Shilyansky

**Affiliations:** 1 Department of Surgery, University of Iowa Hospitals and Clinics, 200 Hawkins Dr., Iowa City, Iowa, 52242 USA; 2 Department of Pathology, University of Iowa Hospitals and Clinics, Iowa, USA

**Keywords:** Congenital intrathoracic accessory spleen, Thoracic splenule, Splenosis, Bronchopulmonary sequestration

## Abstract

**Background:**

Congenital intrathoracic accessory spleen (CIAS) refers to a developmental anomaly resulting in the presence of splenic tissue within the chest. The differential diagnoses for the resulting mass are pulmonary malformations, or lesions with malignant potential. To our knowledge, only four cases of presumed CIAS have been described in literature to date, and no cases were reported in the United States.

**Case presentation:**

We report on a 14-year-old Caucasian female with a left chest mass discovered incidentally on a CT scan performed following an all-terrain vehicle accident. Following resection, the mass was diagnosed as a CIAS.

**Conclusions:**

From our review of literature, we found that CIAS can pose a diagnostic dilemma as it is rare, difficult to distinguish from pulmonary sequestration, or malignancy, and biopsy is often inconclusive. Resection is required to rule out malignancy and determine the diagnosis. Pediatric thoracic surgeons should consider CIAS in their differential for an intrathoracic mass with an inconclusive biopsy.

## Background

The presence of splenic tissue in the thoracic cavity can be acquired or congenital. Acquired intrathoracic spleen (splenosis) commonly occurs following splenic and/or diaphragmatic injury that results in the seeding of splenic tissue in the chest [1]. In contrast, congenital intrathoracic accessory spleen (CIAS) is the presence of splenic tissue within the thoracic cavity from birth. CIAS is extremely rare, and only four reports exist in the literature. To our knowledge, no cases have been reported from the United States to date [2, 3].

In clinical practice, CIAS may pose a diagnostic dilemma [3]. Furthermore, in cases of hematologic disorders such as idiopathic thrombocytopenic purpura (ITP), a missed splenic tissue such as CIAS may result in relapse [3]. As such, we sought to highlight an interesting case of CIAS, and raise awareness about crucial aspects of its presentation based on a review of the literature.

## Case presentation

**Fig. 1:**
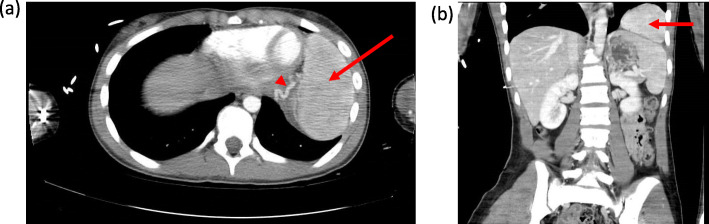
Transverse (**a**) and frontal (**b**) CT scan views of left chest mass in our 14 year old patient. Arrows pointing at the intrathoracic accessory spleen. Arrow head showing inferior phrenic artery blood supply

The patient was a 14-year-old healthy female referred to us after an intrathoracic mass was incidentally discovered in the left hemithorax on a CT scan (Fig. 1) obtained in the setting of a roll-over all-terrain vehicle (ATV) accident. She had no history of previous injuries or hospitalizations. She denied any previous history of symptoms that could be related to the mass, including no shortness of breath or chest pain. Her family history was noncontributory, and physical exam was unremarkable. Contrast enhancement on CT demonstrated an approximately 9.7 × 6.3 cm mass. Blood supply appeared to originate from the left internal mammary artery and the inferior phrenic artery. The left inferior phrenic vein appeared enlarged. Lungs, pleura, esophagus, and chest wall were without abnormalities. No lymphadenopathy was noted. Due to the large size of the mass, and diagnostic uncertainty we recommended resection. Differential included sequestration, malignancy and accessory spleen.

She underwent a left sided video assisted thoracoscopy surgery (VATS). A total of four ports were required -5 mm port in the left 5th intercostal space along the anterior axillary line, 12 mm port in the eighth intercostal space in the mid axillary line on the left (subsequently extended into a 5 cm mini-thoracotomy by dividing the intercostal muscles and latissimus dorsi for retrieval of the specimen), 5 mm port in the third intercostal space in the anterior axillary line, and 5 mm port in the fourth intercostal space in the posterior axillary line. The mass was extra-pleural and in the medial inferior portion of the left thoracic cavity. A LigaSure™ device was used to release the pleural attachments and adhesions along the borders of the mass. Small arterial and venous tributaries were encountered on the anteromedial surface heading towards the internal mammary vessels along the anterior border of the pericardium, as well as, inferomedial arterial vessels headed towards the diaphragm. All vessels were ligated using 5 mm hemoclips and the LigaSure™. Following this, the specimen was dissected fully from the extra-pleural space, the specimen was placed in a specimen bag, partially morcellated and removed through the 12 mm port incision that was extended into a 5 cm mini-thoracotomy by dividing the intercostal muscles and latissimus dorsi. A portion of the sample was sent for frozen section analyses and pathology returned as “Lymphoid and spindle cell lesion, no overt features of malignancy”.

A 20-French chest tube was placed intraoperatively, all port sites were closed, and she was extubated to room air in stable condition. Her hospital course was uneventful. Briefly, chest tube was discontinued on postoperative day (POD) 1, and she was discharged later that day. She was seen again in clinic 2 weeks following discharge and was doing well. Chest X-ray was unremarkable and we arranged for follow up as needed. She continued to do well without concerns 5 months following discharge.

## Histology

**Fig. 2:**
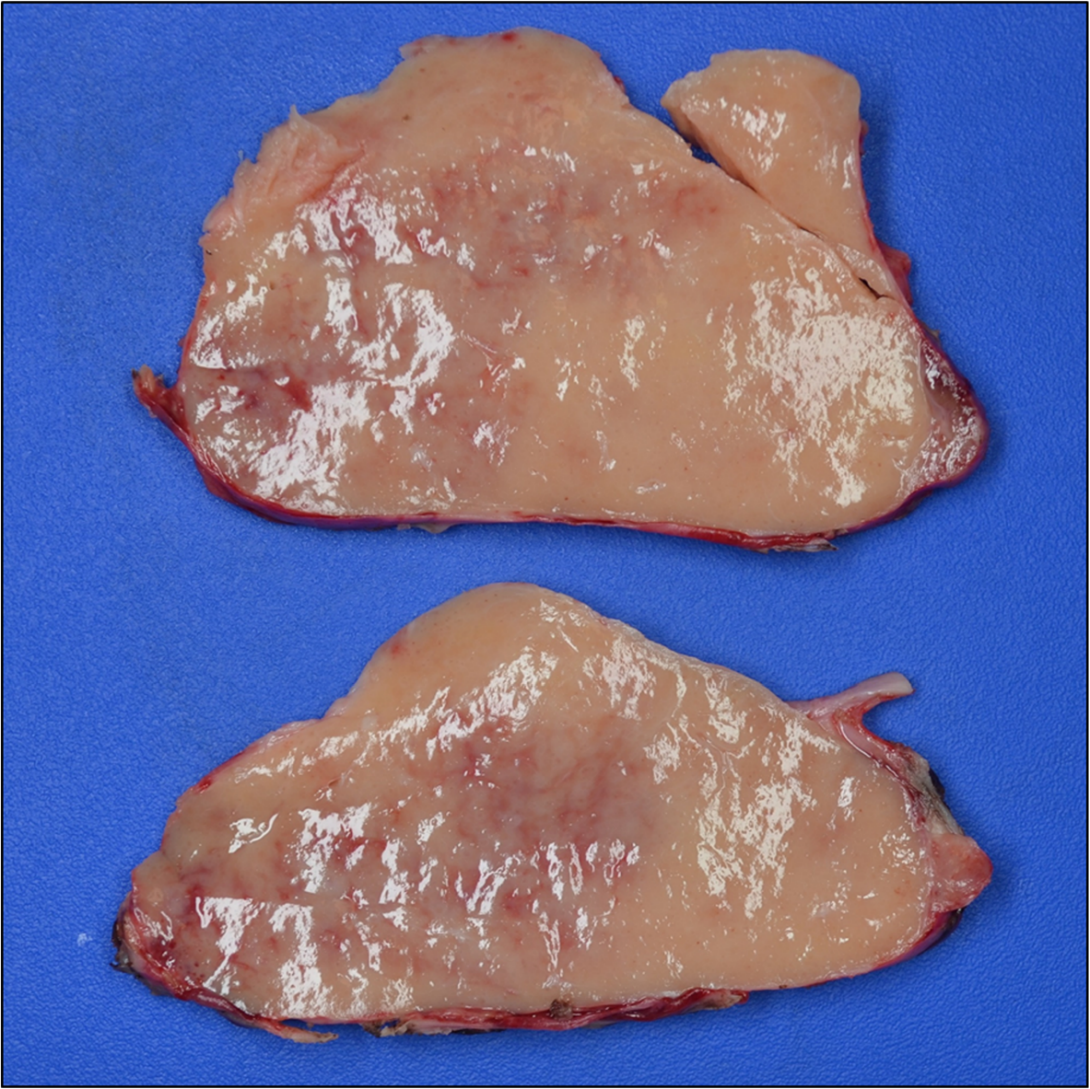
Representative sections of intrathoracic mass which grossly weighed 169 g, measured 13 × 9.5 × 5.6 cm in aggregate, and had firm tan-pink cut surfaces

**Fig. 3:**
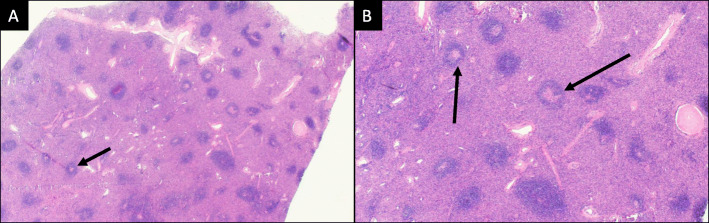
Histologic representation of the intrathoracic mass at 40x (**a**) and 100x (**b**) magnification, H&E stain. There is a lymphoid proliferation with condensation of small lymphocytes around arteries (arrow), the splenic white pulp, and an intervening reticular network of connective tissue and sinusoids, the splenic red pulp

**Fig. 4:**
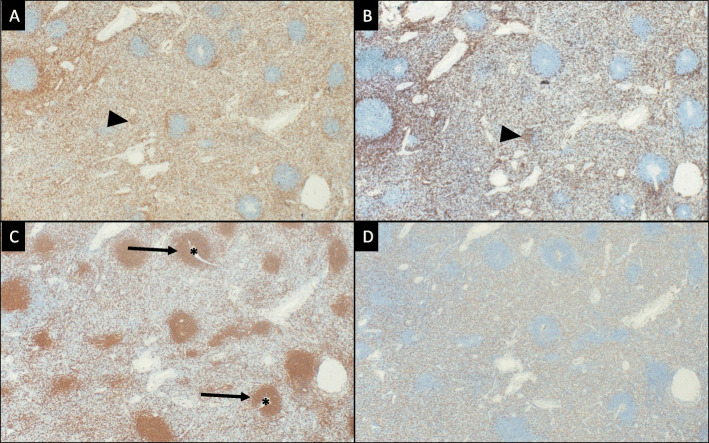
Immunohistochemistry analysis of the intrathoracic mass revealed there to be a population of CD3+ (**a**) T-lymphocytes (arrow head), a subset of which were CD8+ (**b**), in the red pulp and a predominance of CD20+ (**c**) B-lymphocytes (arrows) clustered around the vessels (asterisks) in the white pulp. CD163 (**d**) highlighted the cells lining the sinusoids. For each image, referenced cells are stained brown with all other background cells staining blue. All images are 40x magnification

The specimen was sent to pathology for examination and was grossly described as a 169-g, 13 × 9.5 × 5.6 cm aggregate of solid, tan-pink, firm, and fleshy tissue with smooth cut surfaces (Fig. 2). It had a thin, semi-translucent capsule. Histologic examination revealed a lymphoid proliferation with condensation around arteries consistent with splenic white pulp, as well as, intervening stroma and sinusoids consistent with red pulp (Fig. 3). Immunostains performed on the slides showed an admixture of CD3+ T-lymphocytes (including a proportion of CD8+ T-lymphocytes) within the red pulp and CD20 + B-lymphocytes coalescing around the vessels in the white pulp. CD163 highlighted sinusoidal lining cells (Fig. 4). Flow cytometry was also performed on the specimen and revealed an admixture of T-lymphocytes and B-lymphocytes; no aberrant population was identified.

## Discussions and conclusions

There are only four reports of CIAS to date. Patient characteristics in these cases included: age range of 7 months to 62 years, sex ratio of 3 females to 1 male, and laterality ratio of 3 left sided to 1 right sided chest mass (Table 1). Symptoms at the time of diagnosis included chest pain, shortness of breath, and cough [2].

**Table 1:** Summary of characteristics of cases on congenital intrathoracic spleen. (*M: male, F: female, Asx: Asymptomatic, N: No, Y: Yes, NR: Not Reported; LOS: Length of Stay; SOB: Shortness of breath, VATs: Video-Assisted Thoracoscopic Surgery)*

Author	Age	Sex	Remote hx of trauma	Splenectomy	Laterality	Symptom at diag	Preop diagnosis	Preop workup	Vascular supply	Surgical approach	LOS
Balacumaraswami et al.(2002)	62	F	N	Y (Hx of HS).	Right (paraspinal)	NR	NR	CT; needle biopsy (indeterminate)	intercostal vessels	Right lateral thoracotomy	7d
Lee et al.(2005)	31	F	N	N	Left (lower lobe)	Asx	NR	CT	Abdominal mesenchymal vessels	NR	NR
Lioulias et al. (2007)	47	M	N	N (Hx of thalassemia)	Left (paraspinal)	Chest pain	NR	CT	intercostal vessels	Open mini muscle-sparing left thoracotomy	5d
Bassiony et al.(2012)	7mo	F	N	N	Left (lower lobe)	SOB, Respiratory distress	Sequestration	CT; CT-guided needle biopsy (indeterminate)	From abdomen	Left posterolateral thoracotomy	5d
**Our case**	**14**	**F**	**N**	**N**	**Left (lower lobe)**	**Asx**	**Sequestration**	**CT**	**inferior phrenic and internal mammary**	**Left VATS**	**1d**

Similar to extralobar sequestration, blood supply to CIAS is often extrapulmonary with reports of intercostal vessels, and abdominal mesenchymal vessels being the most common [4]. Sequestration and malignancy were the leading differential diagnoses [2, 4]. As such, CT scan alone may not reliably diagnose CIAS and distinguish it from sequestration [3, 5]. More specific methods of identifying CIAS on imaging include ferumoxide enhanced MRIs, and noninvasive nuclear scintigraphy [3, 5]. Interestingly, in two studies where preoperative biopsies were performed, the pathology was inconclusive [1, 2]. In our case, we performed frozen sections intraoperatively that were also non-diagnostic. In terms of surgical management, all lesions in the left chest were approached with a left incision (VATs or thoracotomy). On the other hand, Balacumaraswami et al. (2002) used a right thoracotomy incision for the right paraspinal splenic mass. All patients were followed up without issue with the longest LOS being 7 days. Our patient was discharged on POD1.

The pathogenesis/embryology of CIAS is unclear [3]. Nonetheless, it is thought to occur between the fifth and seventh weeks of embryologic development secondary to migration of primordial splenic cells into the pleural cavity prior to fusion of pleuroperitoneal fold and esophageal mesentery and septum transversum [1]. This may explain why reports of accessory splenic tissue is almost exclusively on the left [3]. Of note, the only study to date of presumed CIAS to have reported a right sided intrathoracic mass was in a patient whose history was notable for splenectomy at the age of 3 [1]. The presence of the mass in the right paraspinal region may suggest seeding through the hiatus during the splenectomy procedure [3]. CIAS can be differentiated from intrathoracic splenosis as it is almost exclusively a solitary mass that enhances on CT due to its established blood supply. In addition, CIAS are not usually associated with pleural nodules [3, 5].

Taken together, CIAS is a rare and benign presentation which cannot be distinguished from bronchopulmonary sequestration on CT alone. Pediatric thoracic surgeons should consider intrathoracic spleen as a possibility in lesions that have characteristics of sequestration on imaging. Furthermore, biopsies are often equivocal. Resection should be considered if malignancy is suspected, and to decrease the risk of relapse in patients undergoing splenectomy for hematologic disorders [3].

## Data Availability

The datasets used and/or analyzed during the current study are available from the corresponding author on reasonable request.

## Abbreviations

Asx: Asymtomatic
CIAS: Congenital Intrathoracic Accessory Spleen
NR: Not Reported
LOS: Length of Stay
POD: Postoperative Day
VATs: Video Assisted Thoracoscopic Surgery
